# Decoding the drivers of bank erosion on the Mekong river: The roles of the Asian monsoon, tropical storms, and snowmelt

**DOI:** 10.1002/wrcr.20205

**Published:** 2013-04-25

**Authors:** Stephen E Darby, Julian Leyland, Matti Kummu, Timo A Räsänen, Hannu Lauri

**Affiliations:** 1 Geography and Environment, University of SouthamptonHighfield, Southampton, UK; 2 Geography and Environment, University of SouthamptonHighfield, Southampton, UK; 3 Water and Development Research Group, Aalto UniversityAalto, Helsinki, Finland; 4 Environmental Impact Assessment Centre of Finland Ltd.Espoo, Finland

## Abstract

We evaluate links between climate and simulated river bank erosion for one of the world's largest rivers, the Mekong. We employ a process-based model to reconstruct multidecadal time series of bank erosion at study sites within the Mekong's two main hydrological response zones, defining a new parameter, accumulated excess runoff (AER), pertinent to bank erosion. We employ a hydrological model to isolate how snowmelt, tropical storms and monsoon precipitation each contribute to AER and thus modeled bank erosion. Our results show that melt (23.9% at the upstream study site, declining to 11.1% downstream) and tropical cyclones (17.5% and 26.4% at the upstream and downstream sites, respectively) both force significant fractions of bank erosion on the Mekong. We also show (i) small, but significant, declines in AER and hence assumed bank erosion during the 20th century, and; (ii) that significant correlations exist between AER and the Indian Ocean Dipole (IOD) and El Niño Southern Oscillation (ENSO). Of these modes of climate variability, we find that IOD events exert a greater control on simulated bank erosion than ENSO events; but the influences of both ENSO and IOD when averaged over several decades are found to be relatively weak. However, importantly, relationships between ENSO, IOD, and AER and hence inferred river bank erosion are not time invariant. Specifically, we show that there is an intense and prolonged epoch of strong coherence between ENSO and AER from the early 1980s to present, such that in recent decades derived Mekong River bank erosion has been more strongly affected by ENSO.

## 1. Introduction

The Asian monsoon is a key element of the global climate system, playing an integral role in global hydrological and energy budgets [*Clift and Plumb*, 2008; *Cai et al*., 2010]. The monsoon determines the livelihoods and well-being of the region's inhabitants (∼60% of the global population) through its role in controlling amounts of precipitation and, particularly, through attendant impacts on such aspects as drought, flooding, erosion and deposition [*Webster*, 2006; *Clift and Plumb*, 2008]. However, while numerous studies [e.g., *Wang et al*., 2001a, 2001b; *Webster*, 2006; *IPCC*, 2007; *Wang et al*., 2008a, 2008b; *Cai et al*., 2010] have focused on a wide range of aspects of monsoon climate dynamics, investigations of the geomorphic impacts of the Asian Monsoon Climate System (AMCS) on the region's rivers are noteworthy by their relative absence. This is a major gap: five of the world's 15 largest rivers are strongly affected by the AMCS. These “Asian mega-rivers” (the Ganges-Brahmaputra, Yangtze, Mekong (the focus of this study), Pearl and Irrawaddy) play a key role in global biogeochemical cycles, delivering very large (collectively these rivers contribute ∼14% of the total global sediment flux [*Milliman and Syvitski*, 1992]) volumes of sediment to the oceans. The nutrients associated with sediments that are deposited within these rivers and on their floodplains support ecosystem services that sustain high riparian population densities (many of whom are vulnerable poor). Understanding the relationship between climate and sediment transfer processes is, therefore, a significant issue [*Wang et al*., 2011].

In undisturbed systems, climate is a key controlling factor influencing the sediment flux sediment delivered to the oceans [*Milliman and Syvitski*, 1992; *Syvitski and Milliman*, 2007]. A priori it is, therefore, reasonable to postulate that the delivery of fluvial sediment from the Asian mega-rivers is linked to the changing strength of the AMCS [*Wang et al*., 2011]. However, on large rivers considerable fractions of the sediment derived from catchment erosion are stored within their extensive floodplains. A potential consequence of storing such large volumes of sediment is that autogenic geomorphic processes such as channel avulsion may dampen the relationship between climate and net sediment efflux [e.g., *Schumm and Parker*, 1973; *Womack and Schumm*, 1977; *Jerolmack and Paola*, 2010]. It follows that efforts to link sediment yield to climatic controls should ideally consider the role of climate in modulating the individual components of alluvial sediment budgets. However, while the intensity of the AMCS is known to affect catchment erosion rates [*Colin et al*., 2010; *Korup et al*., 2010; *Li et al*., 2010], the relationship between climate and the key geomorphic processes (floodplain deposition and bank erosion) that dominate alluvial sediment budgets has not yet been the focus of significant attention. Some recent work has employed innovative geochronological techniques to resolve floodplain sedimentary records at sufficiently high (near annual) temporal resolution to identify hydro-climatological controls on rates of floodplain sedimentation for large rivers. For example, *Aalto et al*. [2003] used ^210^Pb dating to show that Amazonian floodplain accretion is dominated by episodic events caused by rapidly rising floods, which in turn are associated with cold phase ENSO (La Niña) events. However, while some studies have quantified the time-averaged contribution of bank erosion to the alluvial sediment budget of large rivers [e.g., *Dunne et al*., 1998; *Aalto et al*., 2008], this work has afforded little insight into the climatic events that affect bank erosion. This is primarily because the temporal resolution of the image analysis techniques typically used to quantify rates of channel migration within these sediment budgets is too coarse (typically ∼10^1^ years) to resolve individual storm runoff and associated bank erosion events.

If an attempt to link hydro-climatological variability and change to bank erosion response at high (interannual) temporal resolution and over multidecadal periods represents a novel scientific challenge, then it is also one of considerable significance. The reworking of sediments stored within floodplains contributes significant fractions to alluvial sediment budgets. Moreover, particularly on the Asian mega-rivers, where riparian population densities are amongst the highest in the world, and high levels of poverty enhance the vulnerability of those populations to environmental change, even modest amounts of bank erosion causes serious social and economic consequences. A deeper understanding of how past climate variability and change affects bank erosion is, therefore, required to aid our ability to predict and manage the impacts of future hydro-climatological change.

In this paper, we quantify how historical (last ∼80 years) changes in runoff have impacted bank erosion at distinct sites on the Mekong River. To the best of our knowledge, no prior study has investigated how different components (notably snowmelt, tropical cyclones and monsoon intensity) of regional hydro-climate contribute to variations in bank erosion on one of the world's great rivers. The paper is structured as follows. In section 2, we review the Mekong's hydro-climate before describing the bank erosion model employed herein in section 3. This bank erosion model is forced using historical discharge data to reconstruct multidecadal time series of bank erosion. Analysis of these time series allows us to define a new hydrological index that is pertinent to the problem of estimating annual bank erosion (section 4.1). Subsequently (section 4.2.1), we use hydrological modeling to estimate the individual contributions of snowmelt and tropical cyclones to the Mekong's flow regime. This information is then used to isolate the relative contributions of snowmelt (section 4.2.2), tropical cyclones (section 4.2.3) and monsoon intensity (section 4.2.4) to bank erosion. In section 5, we synthesize our results to show how different elements of regional climate affect bank erosion on the Mekong.

## 2. Hydro-Climatology of the Mekong River: Description and Hypothesis Formulation

### 2.1. Study Site Description

The Mekong river is a globally significant watercourse, ranking 27th in terms of its basin area (816,000 km^2^) [*Kummu*, 2008] and, at approximately 4900 km [*Liu et al*., 2007], 12th in terms of length. The tropical monsoonal climate generates a mean annual runoff of 475 km^3^ [*Mekong River Commission (MRC)*, 2005] and a mean annual sediment load of 1.6×10^8^ t [*Milliman and Meade*, 1983], values that rank tenth and ninth, respectively, amongst the world's rivers. Within Asia the Mekong ranks as the continent's third largest river in terms of sediment load, and its regional significance is highlighted by the point that the Mekong Basin is home to over 70 million inhabitants.

**Figure 1:**
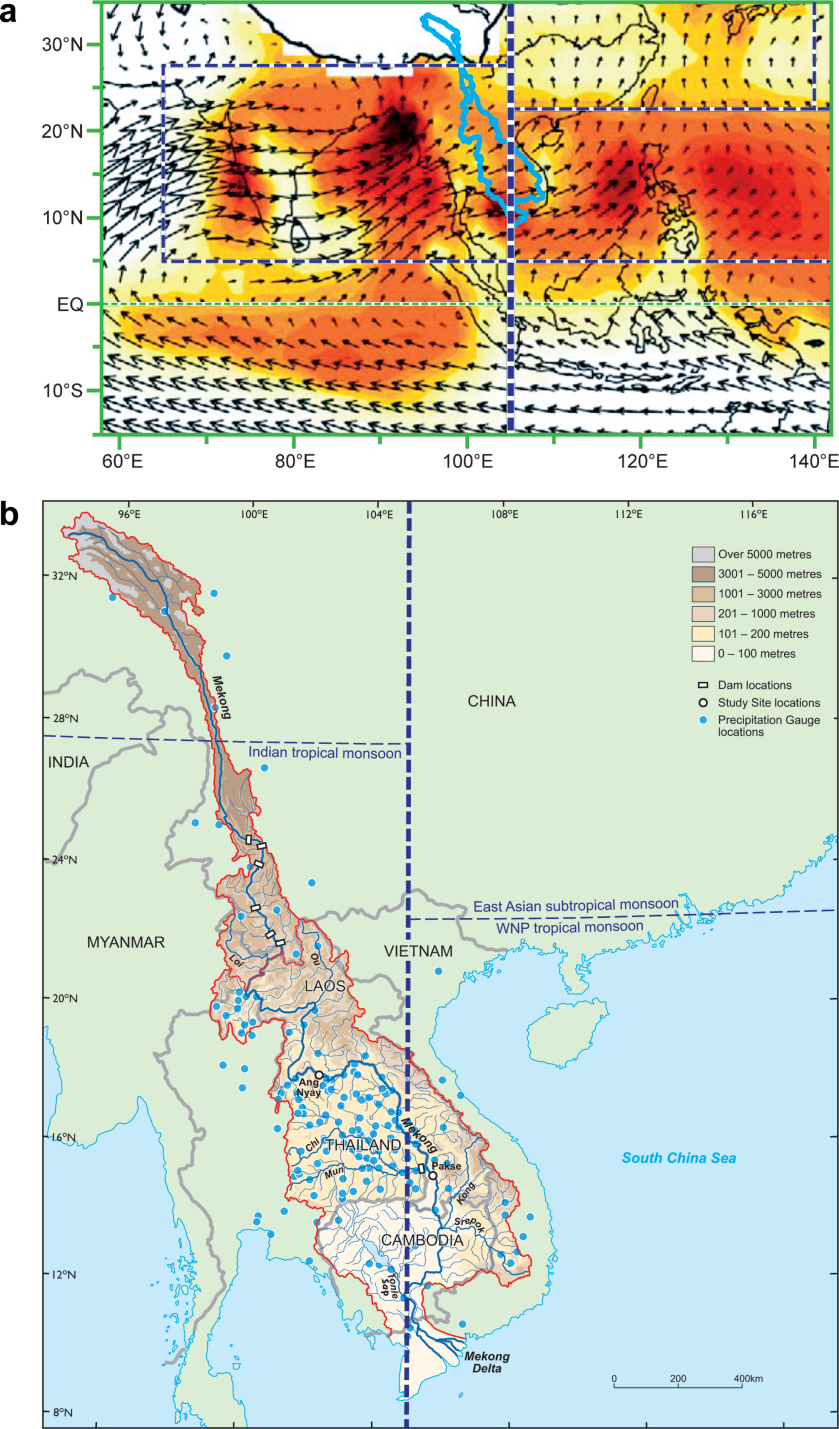
Location of study sites within the Lower Mekong River Basin and their relationship to hydro-climatological controls potentially affecting river bank erosion. (a) Climatological July-August mean precipitation rates (shading in mm/day) and 925 hPa wind vectors (arrows) from *Wang et al*. [2003]. The precipitation and wind climatology are derived from CMAP [*Xie and Arkin*, 1997] (1979–2000) and NCEP/NCAR reanalysis (1951–2000) data, respectively. The three boxes define major summer precipitation areas of the Indian tropical monsoon (5°–27.5°N, 65°–105°E), Western North Pacific (WNP) tropical monsoon (5°–22.5°N, 105°–150°E), and the East Asian subtropical monsoon (22.5°–45°N, 10°–140°E). Note the location of the Mekong Basin at the boundaries of these systems. (b) Detailed map of the Mekong Basin showing the locations of the Ang Nyay and Pakse study sites and major dams constructed since 1993. The locations of meteorological stations used in this study are also indicated.

Similar to other large rivers, its great size means that the Mekong crosses different climatic zones, producing a complicated hydrology. Rising in Tibet and discharging into the South China Sea, the Mekong (Figure 1) can be divided into two units: (i) the Upper Mekong Basin (UMB), which lies within China, and (ii) the Lower Mekong Basin (LMB) to the south of the border between China and Laos. This division demarks the rapid broadening of basin form as it debouches from the confines of the Himalayas, prior to being joined by numerous, sizeable tributaries [*Carling*, 2009]. In the UMB, river flows are fed mainly by spring snowmelt and channels receive only a minor proportion of monsoon precipitation [*Delgado et al*., 2010]. In contrast, runoff in the LMB is dominated by the tropical monsoonal climate [*MRC*, 2005], with additional contributions derived from precipitation associated with tropical cyclones. River flows are therefore variable, with a prolonged annual flood (usually between June and November) and pronounced dry season (December to May) low flows. At Vientiane (mean annual flow of 4500 m^3^/s), discharge varies between mean annual minima and maxima of about 1000 m^3^/s and 16,750 m^3^/s, respectively. Further downstream, at Pakse, substantially increased flows range between 1700 m^3^/s and a mean annual peak of 37,700 m^3^/s, the mean annual flow being 9860 m^3^/s.

While it is dominated by the monsoonal climate, it is noteworthy that the LMB lies at the intersection of three discrete components of the broader AMCS, namely the Indian Monsoon (IM), the East Asian Monsoon (EAM), and the Western North Pacific Monsoon (WNPM) [*Delgado et al*., 2010; *Xue et al*., 2011]. The Lower Mekong can, therefore be divided into three reaches; the differences between each being based on differences in the flood generation mechanism during the monsoon season [*Delgado et al*., 2010; also see Figure 1]:

(i) The reach between the Chinese border to the beginning of the eastern highlands (Annamite Mountains) on the Laos-Vietnam border (i.e., from approximately the Chinese border to the stream gauge at Vientiane) is fed mainly by moisture from the Bay of Bengal and is, therefore, likely to be most strongly affected by the IM

(ii) The reach between Vientiane to Kratie, wherein runoff is fed mainly by orographic precipitation from air masses that are forced by the WNPM and EAM and which cross the eastern highlands, and;

(iii) The reach between Kratie and the Mekong Delta (not considered in this study), which is fed by the same source of moisture as the second, but which lies in much flatter terrain.

In this study, we focus on two study sites on the Mekong main stem in Laos, the first located at Ang Nyay (18°3′15.9″N 102°19′5.5″E), just upstream of the gaging station at Vientiane, and the second at the Pakse gaging station (15°5′55″N 105°47′58″E). The Ang Nyay study site is primarily affected by the IM (i.e., it is located within reach (i) in the list above), whereas Pakse is dominantly affected by the EAM (i.e., it falls within reach (ii)). Clearly, the association of the Ang Nyay and Pakse study sites as being IM and EAM dominated, respectively, is an idealization. For example, the boundaries of the monsoonal systems illustrated in Figure 1 are based on long-term means and in any given year either site can be dominated by either component of the AMCS. In addition, both sites are influenced by additional complicating factors that affect their hydrology, including (a) the base flow component of the flow as derived from snowmelt in the UMB, as well as (b) precipitation inputs derived from tropical storms tracking from the Western Pacific into the interior of the Mekong basin. Finally, insofar as a significant fraction of the runoff at Pakse (∼45%) is derived from upstream sources that also affect Vientiane, the monsoonal runoff regime at Pakse is *not* exclusively affected by the EAM, but rather it is influenced both by the EAM and the IM, as well as the additional factors noted above. Nevertheless, the Ang Nyay and Pakse study sites are, respectively, located upstream and downstream of a key hydrological “hinge point” within the Mekong River [*MRC*, 2005].

### 2.2. Aims, Objectives, and Specific Hypotheses

Our aims are to (i) identify the relative importance of the different contributions of each component of the Mekong's runoff regime (i.e., runoff derived from snowmelt, monsoonal precipitation, and precipitation delivered by tropical cyclones, respectively), and how these change as a function of time and location within the LMB, and (ii) evaluate how these variations affect river bank erosion. Based on the model of the Mekong's hydro-climate set out in section 2.1, we postulate that:

(1) The contribution of snowmelt to bank erosion will reduce with distance downstream (i.e., the snowmelt contribution to bank erosion is greater at Ang Nyay than Pakse);

(2) The contribution of tropical cyclones (TCs) to bank erosion will increase with distance downstream (i.e., the TC contribution to bank erosion is greater at Pakse than Ang Nyay);

(3) At both study sites bank erosion is forced dominantly by monsoon intensity, albeit with a shift in dominance between the IM and EAM at Ang Nyay and Pakse, respectively;

## 3. Simulating Historical Bank Erosion Rates on the Mekong River

The bank erosion model developed by *Darby et al*. [2010] (herein termed D10) is used to reconstruct historical rates of river bank erosion at Ang Nyay and Pakse. Note that the D10 model has been applied previously to these two study sites, albeit for different purposes, and the reader is referred to *Darby et al*. [2010] for full details of the model's theoretical basis, its parameterization, as well as its validation. However, a summary is provided here so that this paper can stand alone. It is well known [e.g., *Thorne*, 1982; *Rinaldi and Darby*, 2007] that bank retreat is driven by combinations of fluvial (particle-by-particle) erosion and mass-wasting (bank collapse under gravity) processes, but in the D10 model a simplifying assumption is made that the rate of bank-top retreat is identical to the rate of fluvial erosion simulated at the bank *toe*. This assumption is justified via the widely accepted concept of basal endpoint control [*Thorne*, 1982; *Rinaldi and Darby*, 2007]: mass wasting is triggered by fluvial undercutting, thus the overall rate of bank retreat is ultimately controlled by fluvial erosion at the toe. The D10 model further assumes that once bank material is eroded it is transferred immediately to the river channel (without entering temporary storage at the bank toe), and that the effects of bank vegetation on erosion can be neglected.

In the D10 model, the fluvial bank erosion rate (*ε*) at the toe of the bank is simulated using an excess shear stress function of the form:


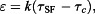
(1)

where the constant, *k*, represents a bank material erodibility coefficient, *τ*_SF_ is the skin drag component of the boundary shear stress, and *τ_c_* is the threshold shear stress required to initiate bank erosion. As outlined below, the threshold shear stress (*τ_c_*) is obtained directly through measurement, while the constant *k* is linked to the threshold shear stress by means of an empirical relationship (see equation (5)). This means that the solution of equation (1) does not involve the use of any additional model calibration parameters [*Darby et al*., 2010].

The D10 model partitions the total boundary shear stress on the eroding river bank into skin drag (*τ*_SF_) and form drag (*τ_d_*) components, the former being the stress relevant for bank erosion:


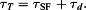
(2)

Correct partitioning using (2) is important because near-bank flows are dominated by the form drag component induced by topographic irregularities associated with eroding river banks [*Thorne and Furbish*, 1995; *Kean and Smith*, 2006a]; indeed the form drag component contributes between 61% and 85% of the total boundary shear stress exerted on the Mekong River banks investigated herein [see *Darby et al*., 2010]. To estimate *τ*_SF_ in (1) D10 employed *Kean and Smith*'s [2006b, 1938] method of partitioning the drag on bank roughness elements. This method assumes that macroscale bank roughness elements take the form of Gaussian-shaped features that protrude into the flow. With this assumption (2) can be expressed as [*Kean and Smith*, 2006a]:


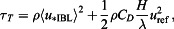
(3)

where *ρ* is the fluid density, u_*__IBL_ is the shear velocity in the internal boundary layer (i.e. the region very close to the bank), *u*_ref_ is a reference flow velocity (see below), *H* and *λ* are geometrical parameters describing the protrusion height and crest spacing of the bank roughness elements, respectively, and the drag coefficient (*C_D_*) is estimated using *Hopson* [1999]:


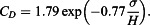
(4)

In equation (4), *σ* is a third bank geometrical parameter which describes the streamwise length scale of the Gaussian bank roughness elements.

*Kean and Smith* [2006a, 2006b] and *Darby et al*. [2010] provide full details of the procedures used to solve (3), but in summary initial estimates are made of the total roughness height and shear velocity, *z_oT_* and u_*T_, in a region of the flow, termed the outer flow region, that is sufficiently distant from the riverbank to be unaffected by wakes shed by bank roughness elements. These initial estimates are made by fitting a logarithmic velocity profile to a (user) specified value of the velocity, *u*_out_, at a point in the outer flow region located at distance *z*_crit_ from the bank. This enables <u_*__IBL_> and *u*_ref_ in (equation (3)) to be determined by means of separate methods within the regions of the near-bank flow affected and not affected by wakes, from which improved estimates of u_*T_ and *z_oT_* are obtained. This iterative sequence is repeated until the solution converges. The input data required to solve (3) includes parameters describing the geometrical characteristics of the bank roughness elements (*H*, *σ*, *λ*), an estimate of the roughness height associated with the skin drag component of the boundary shear stress (*z_o_*_SF_), and a specified flow velocity within the outer flow region, *u*_out_, at a known distance from the boundary, *z*_crit_. Note that, since *u*_out_ varies with distance downstream through a meander bend, the D10 model can implicitly account for variations in bank erosion rate forced by channel curvature. However, the Ang Nyay and Pakse sites employed herein do not exhibit significant curvature.

Full details of the methods used to estimate the bank material erodibility, bank roughness, and outer region flow velocity parameters required to solve equations (1)–(4) are provided in *Darby et al*. [2010], and the parameter values from that study (and used herein) are listed in Table 1. In summary, direct measurements of the critical shear stress, *τ_c_*, of the bank toe materials at the two study sites were obtained using a portable jet-testing device known as a Cohesive Strength Meter [CSM; *Tolhurst et al*., 1999]. Having estimated the value of *τ_c_*, the erodibility parameter *k* was calculated using an empirical relationship (*n* = 83; *r*^2^ = 0.64) derived from jet-testing data obtained by *Hanson and Simon* [2001] (modified here for the units employed in the above):


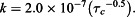
(5)

**Table 1:** Bank Material Erodibility and Bank Roughness Parameters Employed in This Study^a^

	Study Site	
	
Parameter	Ang Nyay	Pakse
*Bank material erodibility parameters*		
*τ_c_* (Pa)	0.83 ± 0.57	0.88 ± 0.47
*k* (m^2^s/kg)	2.20×10^−7^	2.13×10^−7^
Number of samples	6	9
*Bank roughness parameters*		
*H*_reg_ (m)	2.37	3.81
*σ*_reg_ (m)	6.55	5.10
*λ*_reg_ (m)	14.22	22.86
*C_D_*	1.26	1.51
Number of roughness elements	55	44
*z_o_*_SF_ (m)	3.7×10^−4^	1.6×10^−2^

a Symbols as follows: *τ_c_* is the critical shear stress required to initiate river bank erosion (error estimates indicate ± one standard deviation), *k* is a bank material erodibility coefficient, *H*_reg_ is the protrusion height of the bank roughness element, *σ*_reg_ is the streamwise length scale of the bank roughness element, *λ*_reg_ is the crest spacing of the bank roughness element, *C_D_* is the drag coefficient, and *z_o_*_SF_ is the skin friction roughness height. The subscript “reg” refers to the use of the 88^th^ percentile of the distribution of each bank roughness parameter, this value being used to transform the effects of a sequence of irregularly shaped roughness elements into an equivalently rough surface of regularly spaced, identical, elements [see *Kean and Smith*, 2006b; *Darby et al*., 2010].

The bank geometrical parameters *H*, *λ*, and *σ* were estimated through analysis of measured streamwise profiles of bank surface topography; specifically Gaussian curves were fitted to each of the bank roughness elements (“bumps”) identified in the surveyed topographic profiles. For all bumps (99 bumps) statistically significant (*p* < 0.001) fits were obtained, with correlation coefficients ranging between 0.203 (with only four bumps having *r*^2^ values less than 0.500) to 1.0 (*µ* = 0.830, *σ* = 0.142) [*Darby et al*., 2010], meaning that the bank roughness elements observed at the two study sites are approximated well as Gaussian shapes, consistent with the assumptions used in the *Kean and Smith* [2006a] analysis. The skin roughness height parameter, *z_o_*_SF_, was then estimated by analyzing deviations between the measured bank topographic profiles and the Gaussian fits, with *z_o_*_SF_ being approximated by taking a tenth of the standard deviation of the residuals [*Kean and Smith*, 2005; *Darby et al*., 2010].

Values of the outer region flow velocity (*u*_out_) were estimated via acoustic Doppler current profiling (aDcp) surveys. Note that estimates of *u*_out_ were obtained at a specific distance (three times the bank height, *H_b_*) from the river bank (termed the critical distance, *z*_crit_) deemed to be sufficiently beyond the region of the flow affected by wakes shed by bank roughness elements, while not being so distant that local shear stresses are influenced unduly by transverse variations in channel depth [*Darby et al*., 2010]. At Ang Nyay, 10 separate surveys were undertaken at discharges ranging between 1070 m^3^/s and 13,940 m^3^/s, this maximum discharge is 83% of the mean annual peak discharge (1913–2010) of 16,730 m^3^/s observed at Vientiane. At Pakse, data were obtained for 17 separate discharges ranging from 1930 m^3^/s to 28,090 m^3^/s, this maximum value is about 75% of the mean annual peak discharge (37,610 m^3^/s; 1923–2010) for the Pakse gage.

**Figure 2:**
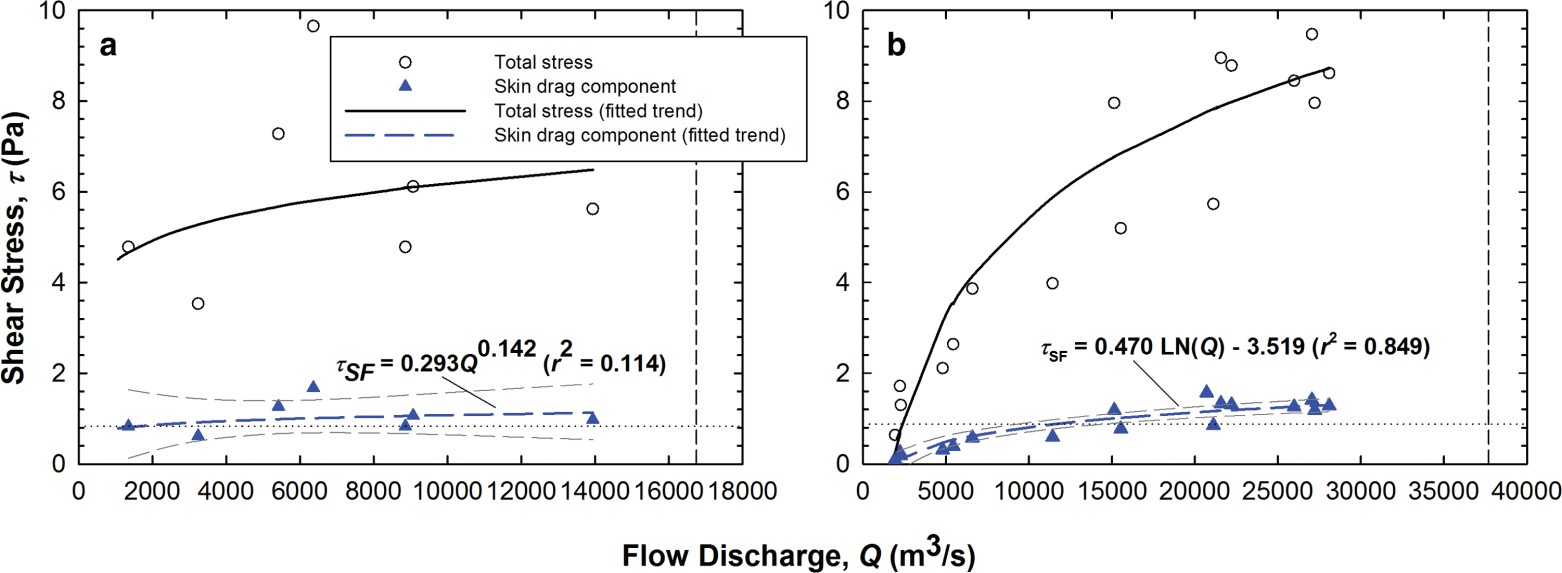
Simulated bank boundary shear stresses as a function of flow discharge for study sites at (a) Ang Nyay, (b) Pakse. The horizontal lines indicate the mean critical shear stress values estimated from CSM measurements (see text for details). Vertical lines indicate the mean peak discharge observed at the gaging stations at Vientiane (for the Ang Nyay study site) and Pakse. The regression relationships (*p* = 0.134 at Ang Nyay, *p* < 0.0001 at Pakse) linking the total stress (solid lines) and skin drag component (dashed lines) of bank boundary shear stress to flow discharge, the latter being used in the bank erosion modeling, are also shown, with the 95% prediction intervals indicated by dashed gray lines.

The significance of the availability of *u*_out_ data for a wide range of discharges is that, together with the bank roughness parameters (Table 1), it enables repeated shear stress partitioning simulations to be undertaken, such that simulated values of total, form and skin drag components of the boundary shear stress are available at each site and across a wide range of discharges. This allows *τ*_SF_ in equation (1) to be expressed as functions of discharge by means of bivariate regression (Figure 2), with *Darby et al*., [2010] obtaining the following relationship for Ang Nyay:


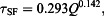
(6)

and Pakse:


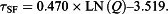
(7)

Note that *Darby et al*. [2010] made no prior theoretical assumption regarding the most appropriate form of fitted function, thus the power and log-linear functions employed for Ang Nyay and Pakse, respectively, simply reflect the superior fits obtained.

Equations (6) and (7) can then be substituted directly into equation (1), together with values of *τ_c_* and *k* (Table 1). Subsequently, historical bank erosion rates are reconstructed by forcing the model with discharge data from the Vientiane (for Ang Nyay) and Pakse flow gaging stations. To assess the predictive performance of the D10 model, *Darby et al*. [2010] compared simulated and observed (as quantified via image analysis of bank-top shifts derived by digitizing aerial photographs and satellite images) rates of bank erosion at four LMR study sites (including Ang Nyay and Pakse), generating a total of six discrete epochs of channel shift during the period 1959–2009. Based on that sample size of six epochs, *Darby et al*. [2010; see Figure 12 therein] found that the D10 model has a general tendency to overpredict erosion, with the root mean square error (RMSE) being 0.53 m/yr. This computed RMSE compares to the mean bank erosion rates of 0.42 m/yr (averaged over the period 1959–2009) and 0.82 m/yr (1959–2000) as observed at Pakse and Ang Nyay, respectively [*Darby et al*., 2010]. Furthermore, at those two sites, which are the specific focus of this study, model simulated rates of bank shift (0.47 m/yr at Pakse; 0.80 m/yr at Ang Nyay) match the observations closely, suggesting the D10 model can be used with confidence.

The lengthy daily discharge records available at the Vientiane (for Ang Nyay) and Pakse stream gages (at time of writing the available data is for the periods 1913–2010 and 1923–2010, respectively) allows the D10 model to be used to reconstruct multidecadal (97 years and 87 years duration for Ang Nyay and Pakse, respectively) time series of instantaneous bank erosion rates at daily time steps. Such time series are sufficiently lengthy to support the analysis of long-term trends in the Mekong's flow regime that might, in turn, be forcing changes in the rates at which the Mekong's river banks are eroding.

## 4. Results

### 4.1. Reconstructed Rates of Bank Erosion on the Lower Mekong River

**Figure 3:**
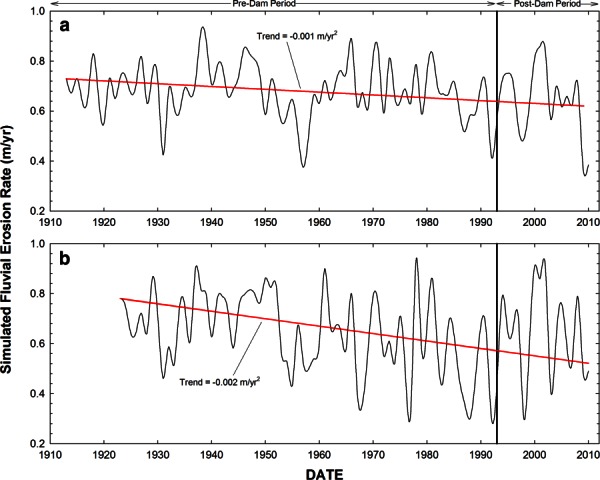
Time series of simulated annual bank erosion rates (solid black lines) for the study sites at: (a) Ang Nyay, (b) Pakse. The solid red lines highlight statistically significant (Mann-Kendall test) declines in bank erosion over the study period. See text for details of trends in the pre-dam and post-dam periods, highlighted on the figure by the vertical solid line (at 1993).

The D10 model was used to simulate bank erosion rates at Ang Nyay and Pakse using the parameter values as listed in Table 1 and the daily discharge values recorded at the stream gauges at Vientiane and Pakse, for the periods 1913–2010 and 1923–2010, respectively. The daily rates of bank erosion obtained for the full time series were then integrated across each calendar year to obtain annual series of simulated bank erosion rates for the full length of the historical flow gaging record (Figure 3). Mean (averaged over 1913–2010 and 1923–2010) annual rates of bank erosion so computed are 0.68 ± 0.11 m/yr and 0.64 ± 0.16 m/yr (error bars indicate one standard deviation) for Ang Nyay and Pakse, respectively.

Nonparametric Mann-Kendall tests [*Kendall*, 1938] reveal significant declines in simulated bank erosion over the course of the 20th century, at both Ang Nyay (1913–2010; *p* < 0.10) and Pakse (1923–2010; *p* < 0.05). The rates of decline (as quantified using nonparametric Theil-Sen regression [*Theil*, 1950; *Sen*, 1968]) are, however, small (0.001 m/yr^2^ and 0.002 m/yr^2^, respectively). Unlike many other large Asian rivers, there have not yet been significant changes in land cover within the Mekong Basin [*Walling*, 2008; *Carling*, 2009; *Kummu and Varis*, 2007; *Kummu et al*., 2010; *Xue et al*., 2011]. However, the UMB has been affected by the construction of a cascade of six dams within Yunnan, the largest and first of these (Manwan) being closed in 1993 (Figure 1b). In addition, the Pak Mun dam, located near the outlet of the Mun River, the largest of the LMR's west bank tributaries and which flows into the LMR just upstream of the Pakse gaging station (Figure 1b), was completed in 1994. To check whether the Yunnan and Mun dams might have influenced the trajectory of simulated bank erosion, we segregated the bank erosion time series into predam and postdam periods (Figure 3) and repeated the Mann-Kendall tests. However, we found no evidence of statistically significant changes in bank erosion trends in the predam versus postdam epochs. This is unsurprising in that, as of 2005, the total active dam storage is only 0.6% and 1.8% of the mean annual flow volume at Vientiane and Pakse, respectively [*Kummu et al*., 2010]. Consequently, the long-term declines in simulated bank erosion rates evident in Figure 3 are most likely forced by changes in the Mekong's hydro-climate. This interpretation is consistent with prior empirical studies that have detected small declines in annual flood maxima trends at stations throughout the Lower Mekong [*Delgado et al*., 2010], and with model simulations [*Kiem et al*., 2008] that predict significant future (2080–2099) declines in the magnitude of the maximum flood under the SRES A1B [*SRES*, 2000] GHG emissions scenario. However, both *Delgado et al*. [2010] and *Kiem et al*. [2008] show that the Mekong's flow regime is becoming more variable with increasingly common flood magnitude extremes, even as the average magnitude of floods declines.

#### 4.1.1. A Hydrological Index of Bank Erosion

**Figure 4:**
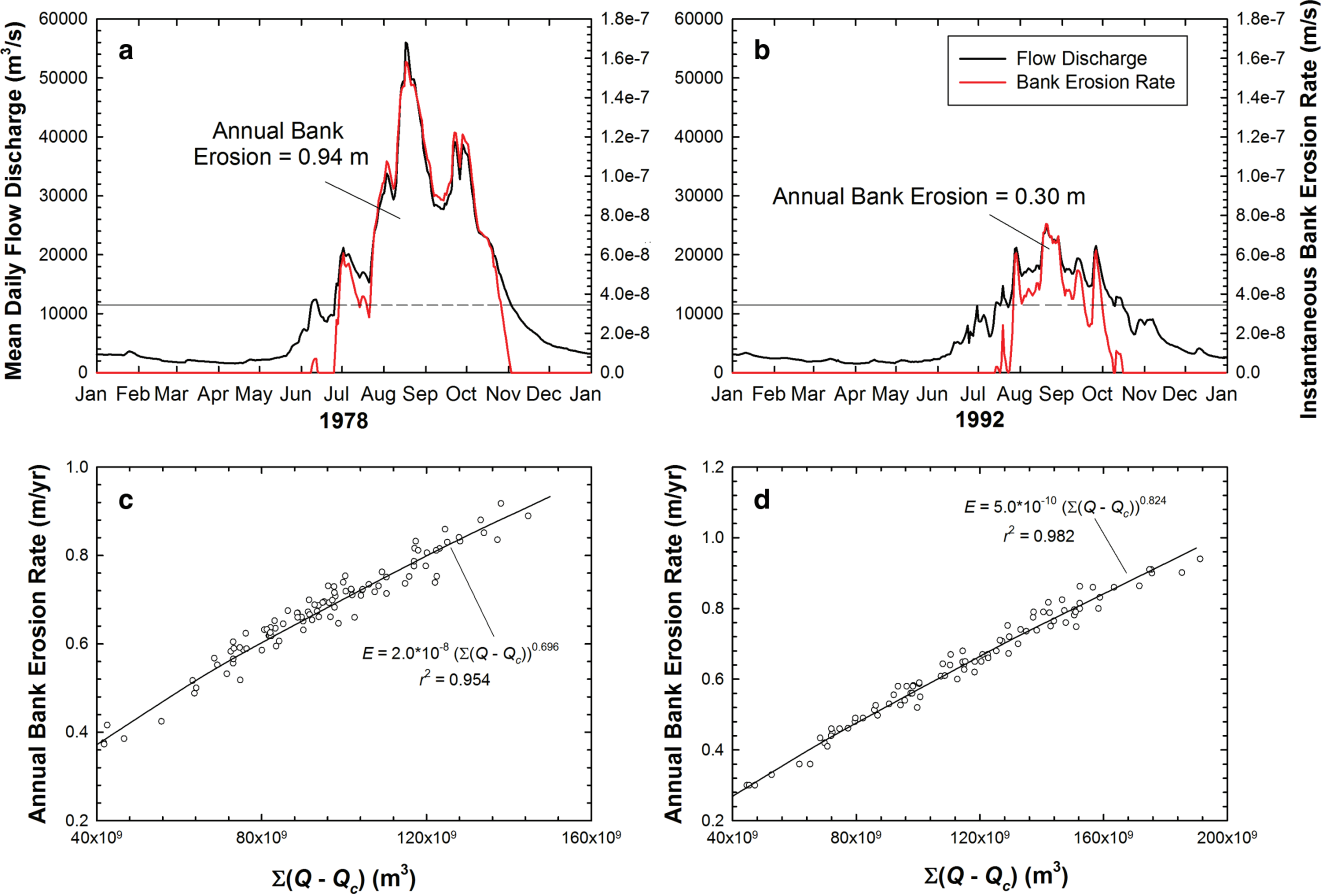
Examples of instantaneous simulated bank erosion rates (red lines) at the Pakse study site induced by selected annual flood hydrographs (black lines): (a) 1978, a high-flow year (the peak discharge of 56,000 m^3^/s being the highest on record) that forces the highest annually integrated rate of bank erosion (0.94 m) in the simulation period 1923–2010; (b) 1992, a low-flow year with the corresponding lowest annually integrated rate of bank erosion (0.30 m) during 1923–2010. The horizontal lines indicate the threshold flow discharge (*Q_c_* = 11,555 m^3^/s) for the initiation and cessation of bank erosion, the solid portion indicating periods when the flow is below the threshold (no erosion), whereas the dashed portion indicates periods when the flow exceeds the threshold. The volume of runoff contained between the dashed line and the solid curve of the annual hydrograph therefore effectively determines the annual erosion rate. Relationships between the annual accumulated runoff over the bank erosion threshold (Σ(*Q* – *Q_c_*)) and annual bank erosion rate at (c) Ang Nyay and (d) Pakse are also shown.

To explore the possible hydro-climatological drivers of both the variability and gradual long-term decline in simulated bank erosion it is first necessary to develop an appropriate analytical framework. Since simulated bank erosion is forced by observed discharge records (equations (6) and (7)), it follows that interannual variability and/or long-term change in simulated bank erosion is forced by interannual variability/change in the Mekong's flow discharge regime. But what hydrological index is most pertinent to describing how changes in flow regime affect the annual rate of bank erosion? Figures 4a and 4b show that a suitable index is the annual volume of runoff above the threshold discharge (*Q_c_*) required to initiate bank erosion. This parameter may be termed the accumulated excess runoff, being denoted in this paper by the acronym AER and/or the symbol Σ(*Q−Q_c_*) [*Darby et al*., 2010]. A key advantage of the AER index is that it recognizes that geomorphic effectiveness is not exclusively controlled by the absolute magnitude of the imposed flow regime, but rather it also incorporates the effects of bank material resistance as well as the duration of above-threshold flows. This approach is advocated in the seminal paper by *Wolman and Gerson* [1978], with *Costa and O'Connor* [1995] employing a similar index, based on stream power, to explore the geomorphic effectiveness of large floods. The quantity Σ(*Q−Q_c_*) therefore integrates different aspects (e.g., magnitude, duration, frequency) of the Mekong's flow regime into a single parameter that is a good proxy of the annual rate of river bank erosion. Figures 4c and 4d illustrate that the relationships between AER and simulated annual bank erosion at Ang Nyay and Pakse take the form of power functions. Note that the very high correlation coefficients obtained for these relationships and shown on the figures are expected given that the simulated bank erosion is itself forced by the flow discharge (see Figures 4a and 4b, and equations (6) and (7)).

**Figure 5:**
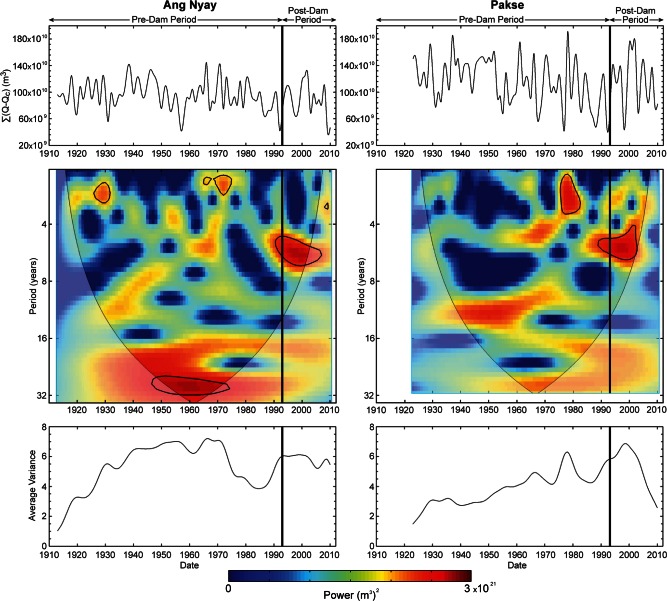
Time series analyses of total accumulated runoff over the bank erosion threshold, Σ(*Q* – *Q_c_*), for the Ang Nyay (left hand column) and Pakse (right hand column) study sites. (top) Time series of Σ(*Q* – *Q_c_*). (middle) Wavelet power spectra of Σ(*Q* – *Q_c_*). Bold contours enclose significant (95% level) times and frequencies; note that the translucent areas indicate the “cone of influence” where edge effects become important and these regions are therefore interpreted with caution. (bottom) Temporal trend of variability in Σ(*Q* – *Q_c_*). Variability is defined here by computing the local “average variance” via integration of the wavelet power spectra shown in the middle panels with respect to the periodicity domain (see text for details). The vertical solid lines denote the division (at 1993) of the time series into pre-dam and post-dam periods.

Interannual variations in AER at the Ang Nyay and Pakse study sites are shown in the top panels of Figure 5. In that same figure, the AER time series are decomposed into wavelet power spectra [*Torrence and Compo*, 2008] to identify the relative power of different periodicities within the AER signals at each point within the time domain (middle panels). As discussed by *Torrence and Compo* [1988], in any analysis of finite-length time series, errors are introduced at the beginning and end of the wavelet power spectrum. These errors are manifest outside the so-called “cone of influence” (indicated on the diagrams by the region of translucent shading), the region of the wavelet spectrum in which edge effects are important. Ignoring low frequencies, and considering zones only within the cone of influence, the wavelet power spectra indicate dominant ∼3 year periodicities in the late 1920s to early 1930s and during the mid-1960s to mid-1970s at Ang Nyay, and during the mid-1970s to early 1980s at Pakse. At both sites, dominant periodicities in the ∼5 year band emerge from around 1990, consistent with studies that postulate a stronger link between (i) the intensity of the EAM and ENSO [*Wang et al*., 2001a, 2001b, 2008a], and (ii) between ENSO and Mekong runoff [e.g., *Xue et al*., 2011] emerging in recent decades.

Following *Delgado et al*. [2010], a nondimensional measure of the local (i.e., for a specific year in the time series) variability in the AER record (here termed the average variance) can be computed by initially normalizing the local wavelet power by the total variance of the AER series and then integrating the normalized wavelet powers with respect to the periodicity scales for the year of interest (i.e., integrating along the vertical axis of the wavelet power spectra). The results (plotted in the bottom panels of Figure 5) show that for Ang Nyay, variability in AER steadily increases from the early part of the 20th century until c.1970, after which variability decreases through to the mid-1980s before increasing again to an approximate “plateau” that coincides with the postdam period. At Pakse, with the exception of an isolated peak in variability during the late 1970s, there is an approximately steady increase in AER variability for most of the 20th century, until a sharp decline commencing in the late 1990s.

Although insights afforded by the wavelet analysis into the dominant periodicities in the AER time series and changes in AER variability over time are useful, linking these periodicities and trends to specific modes of climate change and variability is difficult because the AER parameter is a composite that integrates the effects of three discrete contributions to the Mekong's runoff regime. In the remainder of this paper, we therefore focus on quantifying how the three hydro-climatological drivers of the LMR's runoff regime (snow and glacier melt, monsoon precipitation, and tropical storms) contribute to AER, and hence river bank erosion.

### 4.2. Influence of Tropical Storms, Monsoon Variability and Glacier Melt on AER

#### 4.2.1. Methods: Hydrological Modeling

To isolate the contribution of (i) glacier and snowmelt and (ii) precipitation derived from tropical cyclones to the AER, we employed the VMod hydrological model [*Koponen et al*., 2010]. As implemented for the Mekong, VMod employs a 5 km^2^ grid, with surface elevation, gradient, aspect, vegetation and soil type in each cell being extracted from SRTM DEM [*Jarvis et al*., 2008], GLC2000 land cover [*IES*, 2000] and FAO [*FAO*, 2003] soil-type data sets, respectively. VMod was selected for use here based on its success in prior studies of the Mekong [*Lauri*, 2009] and its subbasins [*MRC*, 2003; *Becker*, 2009; *Räsänen*, 2009]. In summary, VMod is forced with precipitation data, in this case using daily totals estimated from a network of 151 precipitation gauges (Figure 1). Estimates of daily precipitation within each VMod grid cell are obtained by interpolating from the three nearest observations using inverse distance squared weighting. A multiplicative elevation correction (with coefficient 0.0002 mm/m) is employed to account for differences of elevation between each observation point and the location of the grid cell. Note that, prior to 1981, there are insufficient reliable precipitation data to run a basin-wide hydrological model.

VMod simulates snowmelt in each grid cell using a degree-day model, in which the amount of snowmelt is obtained from daily average temperature exceeding a given threshold multiplied by a snowmelt coefficient *K*_melt_. The model also computes snow evaporation, snowpack water storage, and refreezing. Temperature data for each grid cell are again interpolated from the three nearest observation points and corrected for elevation using a temperature lapse rate of −0.006 K/m. The snowmelt parameters were calibrated using flow measurements at the uppermost available flow measurement station (at Chiang Saeng). Glacier melt is computed similarly to snowmelt, albeit using a different set of parameters and the assumption of infinite storage.

**Figure 6:**
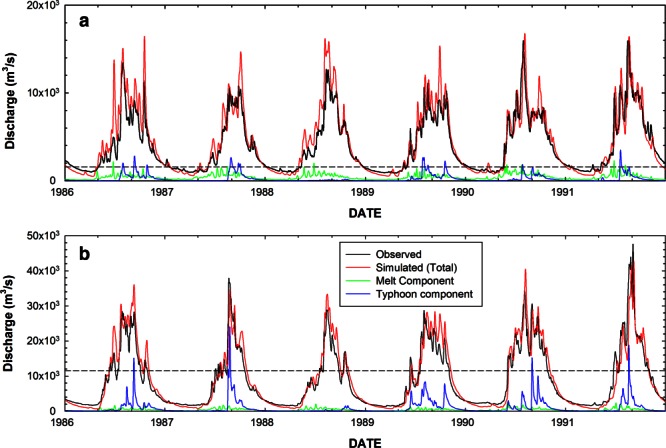
Comparison of VMod simulated (red lines) and observed (black lines) flow discharges at the (a) Vientiane (near the Ang Nyay study site) and (b) Pakse flow gauges on the Lower Mekong River for the period 1 January 1986 to 31 December 1991. The components of the simulated flow attributable to snow and glacier melt (green lines) and tropical cyclones (blue lines) are also shown in relation to the critical flow discharges (horizontal dashed lines) required to initiate bank erosion at each site. Note the difference in the vertical scales of plots (a) and (b).

Figure 6 shows VMod simulated (“baseline” scenario parameterized with data as described above) and observed runoff regimes at Vientiane (close to Ang Nyay) and Pakse. Note that for clarity Figure 6 shows data only for the period 1986–1991, however results for the goodness of fit measures discussed below refer to the entire simulated time series (1 May 1981 to 31 March 2005). We employ three goodness of fit measures to assess the degree to which simulated and observed flows match: (i) the mean discrepancy ratio (Me), which is simply the average of all the ratios (computed at each daily time step) of simulated to observed flows, such that *Me* = 1 indicates perfect agreement between modeled and observed data; (ii) the root mean square error (RMSE), and; (iii) the Nash-Sutcliffe Index (NSI) [*Nash and Sutcliffe*, 1970]. Based on these metrics VMod is, on average, seen to slightly overpredict flow discharge at Vientiane (*Me* = 1.08, RMSE = 1886 m^3^/s), but it under-predicts flows at Pakse (*Me* = 0.90, RMSE = 3106 m^3^/s). Taken together with NSI values of 0.746 and 0.876 at Vientiane and Pakse, respectively, VMod's overall performance can be considered “Very Good” (Vientiane) or “Excellent” (Pakse), based on the classification scheme of *Henriksen et al*. [2008].

**Figure 7:**
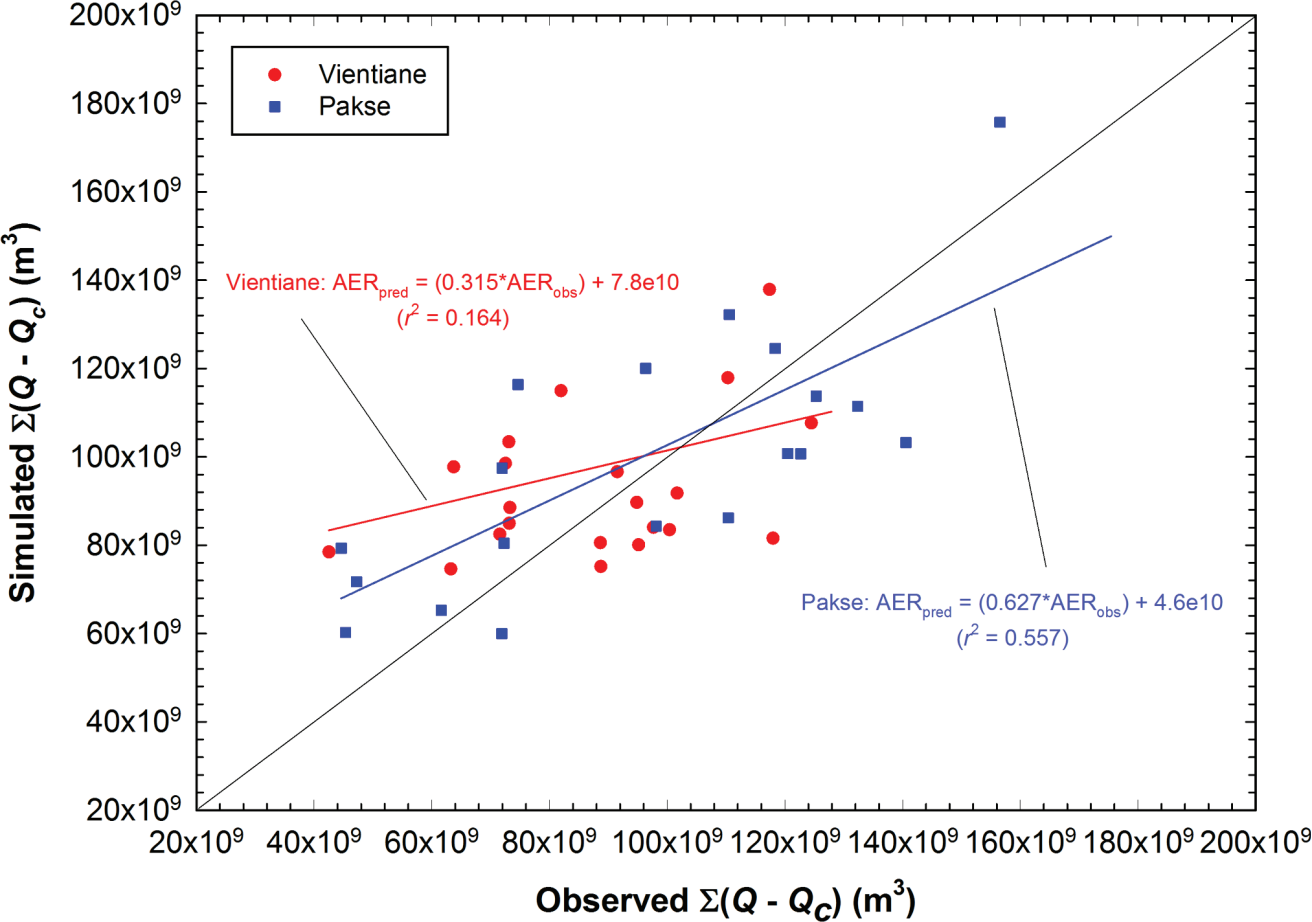
Comparison of simulated and observed total accumulated runoff (AER) over the bank erosion threshold, Σ(*Q* – *Q_c_*), at Vientiane (close to the Ang Nyay study site; denoted by red circles) and Pakse (blue squares). Linear regressions through the data points are also shown (red line for Vientiane, blue line for Pakse) in relation to the 1:1 line of perfect agreement (solid black line).

Of prime importance for the current study is VMod's ability to simulate accurately the higher flows that contribute to AER. Figure 6 indicates that VMod tends to under-predict low flows, but overpredicts peak flows, with mean discrepancy ratios of 1.31 and 1.14 for Vientiane and Pakse, respectively, when considering only the annual maxima. This is confirmed by comparison of simulated and observed AER values (Figure 7) which shows that VMod tends, *on average*, to overpredict AER (*Me* values of 1.25 and 1.01 being obtained for Vientiane and Pakse). Figure 7 shows that there is a cross over at AER values of between about 10.0×10^10^ m^3^ (Vientiane) and 11.0×10^10^ m^3^ (Pakse), with overprediction of AER below these thresholds, but under-prediction of AER otherwise.

VMod was used to isolate the contribution of melt to daily flows (*Q*_Melt_) by differencing model simulated flows obtained in two scenarios. In the first “baseline” scenario, intended to replicate actual conditions, the melt model and parameters were set as described above. In the second, hypothetical, scenario the model was run with melt model parameters set to artificial extreme values that force zero melting. The resulting *Q*_Melt_ series is shown in Figure 6 and discussed in section 4.2.2.

A similar approach was employed to isolate the contribution of tropical cyclones (*Q*_Storm_) to LMR daily flows. In this case, *Q*_Storm_ (see Figure 6 and section 4.2.3) was estimated by differencing the daily flows in the baseline scenario and daily flows as simulated in a scenario in which VMod was forced with precipitation inputs that are revised by removing precipitation delivered by tropical cyclones (TCs) from the baseline precipitation data. We employed the IBTrACS storm tracks database [*Knapp et al*., 2010] to locate TCs and map the paths, at daily time steps, of all recorded storms intersecting or passing near the Mekong Basin during 1981–2005. IBTrACS combines track and intensity estimates for TCs from a range of regional specialized meteorological centres and TC warning centres, as well as other national agencies in a centralized collection [*Knapp et al*., 2010]. The precipitation anomalies associated with the storm paths were defined by first interpolating (nearest neighbor) daily rainfall values observed at the network of 151 stations used in the baseline precipitation scenario onto a 0.1° (∼11 km^2^) resolution grid, thereby providing a surface of estimated observed rainfall covering the entire basin. Next, all precipitation gauges located within a 500 km Haversine search radius [*Rodgers et al*., 2000; *Englehart and Douglas*, 2001] from the centroid of the storm on that date were identified. These identified gauges were then temporarily (for the specific time step) removed from the analysis and an updated precipitation surface (minus the identified gauges) was reinterpolated onto the same 0.1° grid. A precipitation anomaly surface, representing estimated precipitation associated with the identified storm and time step, was obtained by differencing the original and updated surfaces. This process was repeated for each daily time step and observed precipitation series at each gauge were adjusted by subtracting precipitation anomalies within the grid square specific to each gauge from observed daily totals.

We emphasize that in deriving estimates of TC precipitation using the above procedure we have deliberately adopted a method (nearest neighbour interpolation) that is more conservative (i.e., under estimates TC associated precipitation) than some prior studies [e.g., *Englehart and Douglas*, 2001] which simply assume that *all* precipitation within the assigned search radius is TC related. By the same token, while acknowledging that there is uncertainty regarding the typical radii of TCs, our decision to employ a 500 km search radius is again conservative in that it is at the lower end of the range of values typically employed in prior studies [e.g., *Kubota and Wang*, 2009].

#### 4.2.2. Results: Contribution of Snow and Glacier Melt to AER

The mean component of Mekong flow derived from snow and glacier melt (*Q*_Melt_) as estimated from VMod is 9.4% and 4.3% of the mean annual flows passing Ang Nyay (Vientiane) and Pakse, respectively, which can be compared to the winter snow and ice covers of approximately 13% and 7% of the basin areas draining to Vientiane and Pakse [*Kiem et al*., 2005]. Furthermore, Figure 6 indicates that the VMod estimated *Q*_Melt_ values are always less than the threshold discharge values required to initiate bank erosion. By itself, the melt contribution is therefore insufficient to force any bank erosion. Nevertheless, the melt contribution does significantly affect rates of river bank erosion on the LMR. This is because the melt contribution to LMR base flows provides a variable baseline onto which *excess* runoff (i.e., flows above the bank erosion threshold), as generated by monsoonal and cyclone-related precipitation (see below), are superimposed. Thus, even if *Q*_Melt_ values remain below the threshold, the *Q*_Melt_ component still contributes indirectly to the total AER, and hence annual bank erosion.

**Figure 8:**
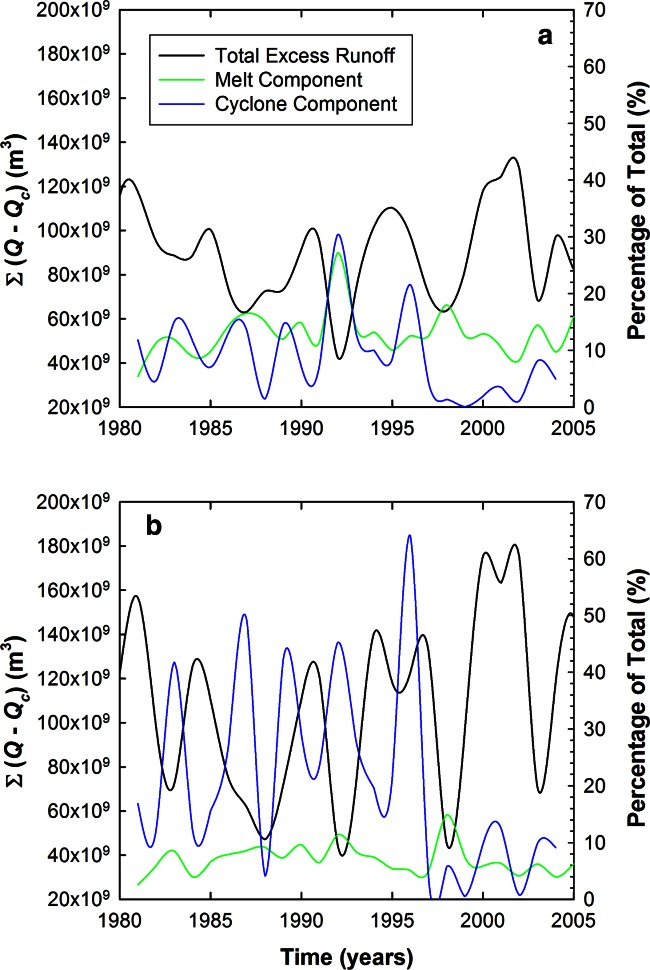
Time series illustrating the proportion of snow and glacier melt (green lines), and cylone-associated rainfall (blue lines), contributing to the total accumulated runoff over the bank erosion threshold (black lines) for (a) Vientiane (close to the Ang Nyay study site) and (b) Pakse during the period 1981–2005.

To quantify the melt contribution, Σ(*Q*_MC_−*Q_c_*), to total AER, *Q*_Melt_ values as estimated from VMod were subtracted from observed daily flows and the (melt removed) time series of excess runoff was reintegrated for each year of record. The resulting Σ(*Q*_MC_−*Q_c_*) time series represents a direct measure of the effectiveness of melt-induced runoff in respect to driving bank erosion (Figure 8). Thus, during the period of available data (1981–2005) the melt component of the total AER varies between 5.4% and 27.2% (Ang Nyay) and between 2.6% and 14.9% (Pakse), with average melt contributions to the total AER of 13.0% and 7.0% at Ang Nyay and Pakse, respectively. Note that it appears to be coincidence that the percentage melt contributions to total AER are identical to the proportions of the snow/ice covered basin areas that drain to the respective gaging stations. These melt components of AER are sufficient to generate significant proportions of the total simulated river bank erosion during 1981–2005: 23.9% of the total bank erosion at Ang Nyay, and 11.1% of the total bank erosion at Pakse, is attributable to melt (Table 2). It is noteworthy that relatively small areas of snow and ice cover in the Upper Mekong Basin (∼13% and ∼7% of the catchment areas upstream of Vientiane and Pakse, respectively) contribute significantly to bank erosion, even at large distances downstream of the snow and ice covered areas. This indicates that relatively small changes in snow and ice cover may have disproportionately large impacts on river bank erosion.

**Table 2:** Overall Proportion of Bank Erosion Forced by Specific Components of the Mekong's Hydro-Climate (1981–2005)

Hydro-Climate Component	Ang Nyay (%)	Pakse (%)
Monsoon precipitation	58.6	62.2
Tibetan snow/glacier melt	23.9	11.1
Tropical cyclones	17.5	26.4

#### 4.2.3. Results: Contribution of Tropical Cyclones to AER

The component of the Mekong's flow derived from TCs (*Q*_ST_) as estimated from VMod is, on average, rather low; accounting for some 5.1% and 7.7% of the total mean annual flow passing Ang Nyay (Vientiane) and Pakse, respectively. Thus, TCs contribute less of the mean annual flow than melt (9.4%) at Vientiane, but more of the mean annual flow than melt (4.3%) further downstream at Pakse. Figure 6 shows that the magnitude of the *Q*_ST_ component is relatively small in relation to the threshold discharge required to initiate bank erosion. However, unlike the *Q*_Melt_ component discussed in 4.2.2 (which never exceeds the threshold discharge), on occasion the *Q*_ST_ component is sufficiently large to exceed the threshold discharge and thus contribute directly to forcing river bank erosion. Figure 6 also highlights the strongly seasonal (summer to early autumn) character of the *Q*_ST_ component flows such that *Q*_ST_ flows are coincident with the summer monsoonal flood pulse. The *Q*_ST_ component flows therefore typically occur during periods when the flow discharge already exceeds the bank erosion threshold, and as such it can be expected that *Q*_ST_ contributes significantly to the total AER.

To isolate the TC contribution, Σ(*Q*_ST_−*Q*_c_), to the total AER, *Q*_ST_ values estimated from VMod were subtracted from observed daily flows and the (storm removed) time series of excess runoff was reintegrated for each year of record. The resulting time series of Σ(*Q*_ST_ − *Q_c_*) is shown in Figure 8. Annual values of Σ(*Q*_ST_ − *Q_c_*) are highly variable, shifting between values of 0.1% and 30.4% (Ang Nyay) and between 0.5% and 64.1% (Pakse), with the average contribution of TCs to the total AER being 9.0% and 21.0% at Ang Nyay and Pakse, respectively. Summary data in Table 2 show that the overall contribution of TCs to simulated river bank erosion as averaged over the period 1981–2005 is 17.5% of the total bank erosion at Ang Nyay, rising to 26.4% at Pakse. It is apparent that there is a cross over in the relative importance of melt versus TCs in forcing river bank erosion between the upstream (Ang Nyay) and downstream (Pakse) study sites.

Tropical cyclones appear to have a disproportionately large impact on bank erosion given that, in the period 1981–2005, TCs delivered between 0.3% and 11.1% of the Mekong's total annual precipitation (average 4.7%) yet account for >25% of the total bank erosion (at Pakse). The reason for this amplification effect has already been mentioned. Since precipitation derived from TCs falls on the Mekong Basin mainly during, or just after, the monsoon season, when the catchment is saturated or near-saturated. Moreover, TC-associated runoff is timed such that it is superimposed on base flows that already exceed the bank erosion threshold. Consequently, TC-associated precipitation is both hydrologically and geomorphologically effective in that the generated runoff contributes directly to generating bank erosion.

#### 4.2.4. Results: Contribution of Monsoon Intensity to AER

The contribution of monsoon precipitation to runoff that is effective in driving bank erosion is the dominant factor forcing bank erosion on the Mekong, accounting for 58.6% and 62.2% of the total simulated bank erosion at Ang Nyay and Pakse, respectively (Table 2). Interestingly, the monsoon-driven component of bank erosion is therefore quite similar in magnitude at both study sites as a result of the downstream trends in melt versus tropical cyclone contributions cancelling out.

## 5. Discussion

### 5.1. Influence of Natural Modes of Climate Variability (ENSO and IOD) on AER

In this section, we consider the extent to which the principal modes of natural climatic variability within the tropical Pacific (El Niño Southern Oscillation, ENSO) and tropical Indian Oceans (Indian Ocean Dipole, IOD) may be linked to AER and hence Mekong River bank erosion. Our focus on ENSO and IOD is justified by the coincidence between their dominant periodicities and the dominant periodicities identified within the AER series (Figure 5). In the case of ENSO, prior studies [*Juneng and Tangang*, 2005; *Xue et al*., 2011] have also demonstrated links between ENSO and specific Mekong flood pulse parameters. Furthermore, rainfall anomalies associated with the IOD over the SE Asia region have also been demonstrated [*Saji et al*., 1999; *Saji and Yamagata*, 2003]. However, to our knowledge no previous study has considered the possible effects of IOD on the Mekong's hydro-climatology, nor has any previous study considered ENSO or IOD links specifically to AER and thus bank erosion.

For ENSO we employ a summer (JJA) ENSO sea surface temperature (SST) index as downloaded from http://jisao.washington.edu/data/globalsstenso/. This index is calculated using input SST data from the *International Comprehensive Ocean-Atmosphere DataSet (ICOADS) version 2.5* for 1800–2007, and the *National Centers for Environmental Prediction near-real-time* (also an ICOADS product) for 2008 to present. The summer ENSO index used here is calculated based on the JJA average of monthly ENSO values determined from the average SST anomaly equatorward of 20° latitude (north and south) minus the average SST poleward of 20°, with anomalies defined with respect to the period 1950–1979. The IOD is characterized in this study using the Dipole Mode Index (DMI), defined as the difference in SST between the tropical western (defined as the box between 50°E–70°E and 10°S–10°N) Indian Ocean and the tropical SE (defined as the box between 90°E–110°E and 10°S–0°) Indian Ocean [*Saji et al*., 1999].

Considering first the entire >80 year period of record, statistically significant (*p* < 0.01) negative correlations between AER and both ENSO and DMI are evident at the Pakse (downstream) study site (Table 3). However, the strength of these correlations is weak, with *r* values of −0.362 (ENSO) and −0.292 (DMI). While no significant correlations are found between total AER and either ENSO or DMI at Vientiane (upstream), the weak (*r* = −0.201) negative correlation between AER and ENSO at Vientiane is almost (*p* = 0.055) significant at the ∼95% confidence level. Although the significant correlations between AER and ENSO and AER and DMI in the downstream portion of the Mekong basin are weak, they are physically plausible in that prior studies have highlighted how cold phase ENSO [e.g., *Xue et al*., 2011] and DMI [e.g., *Saji and Yamagata*, 2003] are associated with increased summer (i.e., monsoon) precipitation over the Indochina peninsula. Indeed, Table 3 shows the strength and significance of the relationships between ENSO, DMI and the three specific components (melt, monsoonal precipitation, and precipitation associated with TCs) of the total AER. As explained previously (see section 4.2.1), these analyses are based on data for the period 1981–2005, reducing the sample sizes used in the correlation analysis. Significant (*p* < 0.05) negative correlations between ENSO and DMI and the monsoonal component of AER are evident at both Vientiane and Pakse during 1981–2005. One other significant (*p* < 0.05) relationship highlighted in Table 3 is the correlation (*r* = −0.456) between DMI and the melt component of AER at Vientiane, with a correlation of slightly reduced strength (*r* = −0.401) further downstream (at Pakse) almost (*p* = 0.052) significant at the 95% level. Although these correlations between DMI and the melt component of AER are only moderate, they are physically plausible because there is a known teleconnection between the IOD and winter precipitation over NE Asia and Tibet [*Saji and Yamagata*, 2003]. In contrast, we find no significant relationship between ENSO and the melt component of the AER, nor do our data reveal any significant correlation between ENSO or IOD and the tropical cyclone component of AER.

**Table 3:** Pearson Correlation Coefficients Showing the Strength of Relationship Between Σ(*Q* – *Q_c_*) and Selected Climatological Indices (ENSO, DMI, IMI and EAMI, See Text for Acronyms and Details) for Ang Nyay (1913–2010 for ENSO and DMI, 1948–2010 for IMI) and Pakse (1923–2010 for ENSO and DMI, 1948–2010 for EAM)^a^

	Pearson Correlation Coefficients Linking Σ(*Q* – *Q_c_*) and its Components with Selected Climatological Indices	
	
Climate Index	Ang Nyay (Vientiane)	Pakse
	*Total Σ(Q – Q_c_)*	
ENSO	−0.201 (*p* = 0.055)	−**0.362** (*p* = 0.0008)
DMI	−0.135 (*p* = 0.198)	−**0.292** (*p* = 0.008)
IMI	0.022 (*p* = 0.871)	n/a
EAM	n/a	**0.424** (*p* = 0.0008)
	*Monsoon component of Σ(Q – Q_c_)*	
ENSO	−**0.591** (*p* = 0.002)	−**0.532** (*p* = 0.007)
DMI	−**0.469** (*p* = 0.021)	−0.398 (*p* = 0.054)
IMI	0.066 (*p* = 0.764)	n/a
EAM	n/a	−0.005 (*p* = 0.982)
	*Melt component of Σ(Q – Q_c_)*	
ENSO	−0.150 (*p* = 0.483)	−0.308 (*p* = 0.143)
DMI	−**0.456** (*p* = 0.025)	−0.401 (*p* = 0.052)
	*Tropical cyclone component of Σ(Q – Q_c_)*	
ENSO	0.359 (*p* = 0.085)	0.216 (*p* = 0.312)
DMI	0.160 (*p* = 0.456)	−0.072 (*p* = 0.740)

a Pearson correlation coefficients linking the melt, cyclone and monsoon components of Σ(*Q* – *Q_c_*) to these climate indices are also shown (1981–2005). Significant (*p* < 0.05) correlations are highlighted in bold text.

The preceding analyses quantify the strength of any relationships between AER and natural modes of climate variability (ENSO and IOD) across the full range of the respective ENSO and DMI indices. However, it is also useful to evaluate whether AER exhibits a more sensitive response to extreme (warm and cold phase) ENSO and IOD episodes. We therefore determined composite AER anomalies for (i) all extreme ENSO events, (ii) all extreme IOD events, as well as (iii) ENSO-independent IOD events, and (iv) the IOD-independent ENSO events (Table 4). These composite anomalies were computed following *Saji and Yamagata* [2003]: an average of the AER anomaly was initially found for all positive (ENSO or IOD) events, then an average was calculated for the corresponding negative (ENSO or IOD) events. The difference between these averages was determined and divided by two to give the composite anomaly.

**Table 4:** Composite Σ(*Q–Q_c_*) Anomalies (i.e., Deviation From Mean Σ(*Q–Q_c_*)) During 1910–2010 for Specific Modes of Climate Variability^a^

Composite Σ(*Q* – *Q*_c_) Anomaly for	Ang Nyay (%)	Pakse (%)
All 28 IOD events	3.3	9.9
17 ENSO-independent IOD events	7.1	14.8
All 40 ENSO events	−6.8	−3.7
29 IOD-independent ENSO events	−4.8	−10.3
11 co-occurring ENSO and IOD events	−3.5	9.9

a Note that composite anomalies are computed following *Saji and Yamagata* [2003] and are expressed as percentages of the observed mean Σ(*Q* – *Q_c_*). Specific ENSO and IOD events are identified following *Meyers et al*. [2007] and *Ummenhofer et al*. [2009].

Composite AER anomalies calculated following the above procedure indicate that the influence of ENSO and IOD events on AER is greater in the downstream portion of the LMR (Table 4), but the overall magnitude of the AER and hence bank erosion response, even to extreme ENSO and IOD episodes, is seen to be rather modest. Specifically, AER (annual bank erosion) anomalies associated with IOD events amount to 7.1% (4.9%) and 14.8% (12.0%) at Vientiane and Pakse, respectively. The AER (annual bank erosion) anomalies associated with ENSO events are still smaller, at −4.8% (−3.3%) and −10.3% (−8.8%) for Vientiane and Pakse. Note that for the highlighted rows in Table 4, ENSO and IOD episodes are treated as independently operating modes of climate variability (see *Saji and Yamagata* [2003] for justification), meaning that co-occurring ENSO and IOD events (there are 11 in the period of interest here) are removed to avoid biasing the results. Two points follow from this treatment. First, we are able to show that, while the effects on AER are modest, (ENSO independent) IOD events nevertheless have a greater (14.3% versus −10.3% in the downstream portion of the drainage basin, at Pakse) impact on AER and hence annual bank erosion rates (12.0% versus −8.8% at Pakse) than (IOD independent) ENSO events. Second, because the direction of the response of AER to ENSO and IOD events is of opposite sense, prior studies of Mekong hydrology that consider only ENSO (and not IOD) must have under-estimated the true magnitude of the ENSO effect, because such studies would have been biased by not isolating co-occurring IOD events.

**Figure 9:**
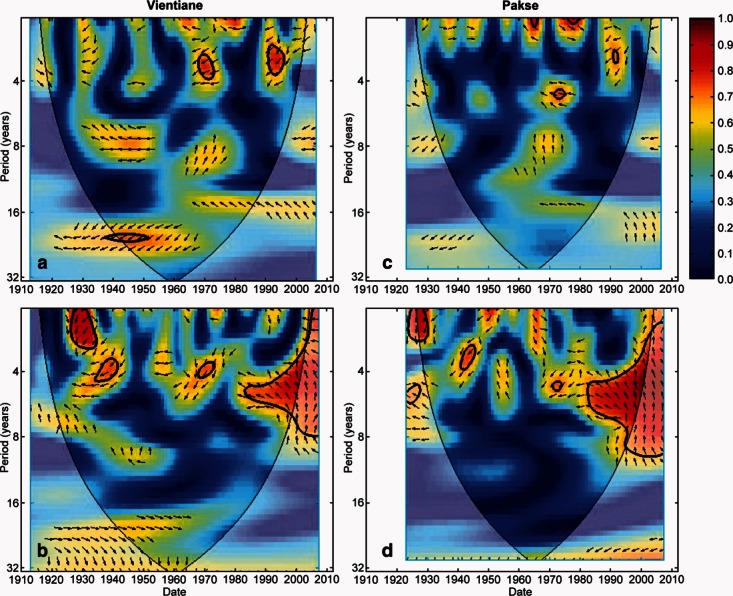
Squared wavelet coherence between AER and climate variability time series of the Dipole Mode Index (DMI) and El Niño Southern Oscillation (ENSO), showing regions in the time-frequency space where these series covary: (a) AER versus DMI at Vientiane; (b) AER versus ENSO at Vientiane; (c) AER versus DMI at Pakse; (d) AER versus ENSO at Pakse. See text for details of the DMI and ENSO indices employed in this study. The color bar indicates the level of significance of coherence between the defined time series, with the 5% significance level against red noise highlighted by solid black contours. The translucent areas indicate the cone of influence where edge effects become important and these regions are therefore interpreted with caution. The vectors indicate the relative phase differences between the two time series, with in-phase pointing right, anti-phase pointing left, and AER leading (pointing straight down) or lagging (pointing straight up) the respective climate indices.

A limitation of the above analyses is that they consider the time-averaged behaviour of ENSO and IOD over a multidecadal period. We therefore employed wavelet coherence analyses [*Torrence and Compo*, 1998; *Grinsted et al*., 2004] to identify the extent to which the relationships between ENSO and AER and between IOD and AER exhibit epochal behaviour. Figure 9 shows that statistically significant (*p* < 0.05) episodes of IOD-AER coherence are indeed apparent during (i) the late 1960s to mid-1970s and (ii) the early 1990s at both Vientiane and Pakse, with epochs of ENSO-AER coherence also evident during (i) the late 1930s to early 1940s, (ii) the late 1960s to mid-1970s (coincident with an epoch of IOD coherence) and, most notably, (iii) an intense and prolonged episode of ENSO-AER coherence from the early 1980s to present. Of these statistically significant links identified by the wavelet coherence analysis, the latter is the most physically plausible in that it may be explained in relation to a change in the relationship between the intensity of the East Asian Monsoon and ENSO, as discussed further below.

### 5.2. Role of Monsoon Intensity

To evaluate whether monsoon intensity may be linked to AER we employ two circulation indices (see below for definitions) proposed by *Wang and Fan* [1999]. While the choice of circulation indices is to some degree arbitrary, we prefer the *Wang and Fan* [1999] indices because (i) they are based on theoretical considerations [see *Wang and Fan*, 1999], and; (ii) they employ circulation data, meaning that data are available for a relatively long period (from 1948 using NCEP-NCAR reanalysis data). Regarding the specific definitions of these circulation indices, *Wang and Fan* [1999] defined the WSI1 (representative of the IM and referred to as such herein) circulation index using the westerly shear (i.e., U850-U200) averaged over the region (5°–20°N, 40°–80°E). In contrast, *Wang and Fan* [1999] argued that an EAM index is best represented by a shear-index (which they termed SSI2), defined using the southerly shear averaged over the combined areas (5°–15°N, 120°–145°E) and (5° S–5°N, 90°–120°E). However, *Wang and Fan* [1999] noted that in this EAM region the 200 hPa wind and 850 hPa wind anomalies are not vertically aligned, meaning that the SSI2 shear index is not adequate. Consequently *Wang and Fan* [1999] constructed an alternative circulation index, DU2 (referred to in this paper as the EAM index) using only the 850 hPa winds, that is the difference between the westerly anomalies averaged over (5°–15°N, 90°–130°E) and the westerly anomalies averaged over (22.5°–32.5°N, 110°–140°E).

Regarding the relationships between the monsoon indices and AER, our data (Table 3) show no link between the IM index and AER (and thus bank erosion) at Vientiane during 1948–2010. However, there is a significant (*p* < 0.001) positive correlation of moderate strength (*r* = 0.424) between the EAM index and AER at Pakse. Note that in Table 3 we do not report correlations for IM versus AER at Pakse, nor EAM versus AER at Vientiane, since we have already (section 2.1) postulated that Vientiane is IM dominated whereas Pakse is EAM dominated. The absence of any correlation between the IM and AER at Vientiane is therefore surprising, but may be explained by the findings of *Wang et al*. [2001a, 2001b, 2008a, 2008b] and *Delgado et al*. [2011] who have documented a strengthening relationship between ENSO and the EAM, along with a weakening relationship between the IM and ENSO. In this regard, in Table 3 it is noteworthy that the correlation coefficients between ENSO and the *monsoon component* of AER segregated out for the more recent (1981–2005) period are much greater than those between ENSO and the *total* AER for the full (>80 years) period of record. It is therefore plausible to suggest that the strong recent (post 1980) ENSO-AER coherence identified in the preceding section is indeed likely caused by a strengthening relationship between ENSO and monsoon precipitation.

## 6. Conclusion

In this paper, we have undertaken the very first detailed evaluation of the possible links between regional climate, climate fluctuations, and river bank erosion response for one of the world's largest rivers, the Mekong. Using a physically based river bank erosion simulation model we initially defined an index, AER, which integrates those characteristics of the Mekong's flood-pulse hydrology that are pertinent to forcing river bank erosion. We then used observational records dating from 1913 (Vientiane) and 1923 (Pakse) to reconstruct variations in AER. Although its hydrology is dominated by the Asian Monsoon, similar to other large rivers, the large scale of the Mekong and its north-south alignment means that it crosses different climatic zones. Consequently, the Mekong's flood hydrology is a complex function of different climatological controls. We therefore used a hydrological model to isolate how three discrete flow generation mechanisms (snow/glacier melt, monsoonal precipitation, and precipitation associated with tropical cyclones), contribute to variations of AER for the recent (1981–2005) period. Our specific findings are summarized as follows:

(1) Hydrological modeling was used to isolate the contributions of melt, monsoonal precipitation and tropical cyclone derived precipitation to AER (and thus bank erosion) on the Mekong River for the period 1981–2005. The proportion of bank erosion attributable to melt (23.9% at Vientiane, declining to 11.1% at Pakse) and tropical cyclones (17.5% at Vientiane, increasing to 26.4% at Pakse) varies spatially, with the proportion attributable to the monsoon remaining relatively constant (∼60%) at both sites.

(2) There have been small but statistically significant (*p* < 0.10 at Vientiane; *p* < 0.05 at Pakse) declines in AER, and hence annual bank erosion rates, at both Vientiane (from 0.72 m/yr in 1913 to 0.39 m/yr now) and Pakse (from 0.78 m/yr in 1923 to 0.50 m/yr now) (Figure 3).

(3) Multidecadal records of AER on the Mekong exhibit high levels of interannual variability. In terms of candidate modes of climate variability driving these changes, our data indicate that IOD events exert a stronger control on AER and hence bank erosion than ENSO events. However, when averaged over the entire multidecadal study period, it is acknowledged that the overall affect of these IOD and ENSO events on bank erosion is small. Specifically, IOD events contribute between 4.9% (Vientiane) and 12.0% (Pakse) of the variation in annual bank erosion, compared to 3.3% (Vientiane) and −8.8% (Pakse) for ENSO events.

(4) Nevertheless, we also find that ENSO in particular exhibits epochal behavior in relation to statistically significant (*p* < 0.05) periods of coherence with AER and hence bank erosion at both Vientiane and Pakse (Figure 9). That is, the generally weak relationships between ENSO and AER and bank erosion highlighted above are not time invariant. Of particular interest is our identification of an intense and prolonged episode of ENSO-AER coherence from the early 1980s to present.

Two implications arise from the above findings. The first concerns the point that IOD events exert a slightly greater control on AER (and hence bank erosion) than ENSO events, yet all prior studies of the Mekong's hydrology have considered only the latter. Notwithstanding the modest magnitude of the influence of both IOD and ENSO on AER, this may be regarded as a limitation, not only because prior studies have neglected IOD's role in affecting regional climate variability, but also because the failure to account for ENSO events that co-occur with IOD events means that the significance of ENSO as quantified in prior studies has been under-estimated. Furthermore, since the IOD's sphere of influence is extensive [*Saji et al*., 1999; *Saji and Yamagata*, 2003], then the role of the IOD in driving natural fluctuations of water and sediment flux on other Asian mega-rivers [e.g., *Wang et al*., 2011] has also likely been overlooked. Consequently, future studies of the hydro-climatological controls on the geomorphic functioning of these large rivers should assess all relevant modes of natural climate variability. The second implication concerns the significance of the tropical cyclone and melt-derived contributions to AER and hence bank erosion on the Mekong River. In both cases significant amounts of AER and thus bank erosion are derived from melt contributions sourced from relatively small areas of catchment snow and ice cover and TC-associated contributions derived from relatively small proportions of annual precipitation, respectively. We therefore speculate that AER and hence bank erosion may be sensitive to relatively minor changes in the proportion of catchment snow and ice and TC-derived precipitation. We suggest that other large rivers with significant proportions of snow and ice cover (e.g., Indus, Brahmaputra), or which are affected by tropical cyclones (e.g., Yangtze, Mississippi, Pearl), can therefore be expected to have bank erosion dynamics that are significantly influenced by these aspects of the climate system.
